# Editorial: The role of adjuvant and neoadjuvant therapy in resectable esophageal cancer

**DOI:** 10.3389/fonc.2025.1688480

**Published:** 2025-09-11

**Authors:** Xiaokun Li, ZhuangZhuang Cong, Yong Qiang

**Affiliations:** ^1^ Department of Thoracic Surgery, West China Hospital, Sichuan University, Chengdu, China; ^2^ Department of Thoracic Surgery, Nanjing Jinling Hospital, Nanjing University, Nanjing, China

**Keywords:** esophageal cancer, neoadjuvant therapy, chemoimmunotherapy, chemoradiotherapy, immunotherapy

According to GLOBOCAN data from 2020, esophageal cancer in China ranks fifth among all malignant tumors in terms of incidence, with as many as 324,000 new cases and 301,000 annual deaths (1). These figures indicate that the burden of esophageal cancer in China is quite heavy, accounting for 55% of global esophageal cancer cases. Unlike Western countries, esophageal cancer patients in China are mainly affected by squamous cell carcinoma, and 40% of patients are already in the advanced stage when they seek medical treatment (2). For patients with locally advanced esophageal cancer, surgery is a key treatment method, but patients with locally advanced esophageal cancer may achieve better clinical outcomes when undergoing surgery after neoadjuvant therapy. However, the prognosis of these patients remains relatively poor. Between 2009 and 2015, the overall 5-year relative survival rate for esophageal cancer was 21.4%. Specifically, the 5-year relative survival rate for local tumors was 46.7%, for regional metastasis it was 25.1%, and for distant metastasis, it was only 4.8% (3).

The CROSS study is a randomized controlled trial with a duration of over 10 years, whose results have clearly demonstrated that neoadjuvant chemoradiotherapy offers a significant survival advantage over surgery alone in the treatment of locally advanced esophageal cancer. According to this study, the absolute survival benefit for patients within 10 years was 13%. Furthermore, the stratified analysis of pathological types conducted in the study indicated that patients with squamous cell carcinoma derived greater benefits from neoadjuvant chemotherapy compared to those with adenocarcinoma. In the squamous cell carcinoma subgroup, the median overall survival of patients who received preoperative concurrent chemoradiotherapy combined with surgery reached 82 months, while the overall survival of the control group that underwent surgery alone was 21 months (4).

In recent years, immunotherapy (anti-PD-1/PD-L1 therapy) has shown broad application prospects in the neoadjuvant treatment of locally advanced esophageal squamous cell carcinoma, and a large number of studies on chemotherapy combined with immunotherapy have followed. The commonly used chemo-immunotherapeutic drugs mainly include paclitaxel or nab-paclitaxel, platinum-based drugs, anti-PD-1 or anti-PD-L1 antibodies. Recently, the phase III clinical study published by Professor Yin Li’s team has landmark significance for neoadjuvant chemoimmunotherapy in locally advanced esophageal squamous cell carcinoma (5). Their study evaluated the efficacy and safety of neoadjuvant camrelizumab plus chemotherapy followed by adjuvant camrelizumab, compared with neoadjuvant chemotherapy alone. A total of 391 patients with resectable thoracic locally advanced esophageal squamous cell carcinoma were enrolled and randomized in a 1:1:1 ratio to receive two cycles of neoadjuvant therapy. The treatment regimens included: camrelizumab + nab-paclitaxel + cisplatin (Cam+nab-TP group, n=132); camrelizumab + paclitaxel + cisplatin (Cam+TP group, n=130); and paclitaxel + cisplatin (TP group, n=129), followed by surgical resection in all groups. The results showed that the Cam+nab-TP and Cam+TP groups exhibited significantly higher pathological complete response (pCR) rates of 28.0% and 15.4%, respectively, compared with 4.7% in the TP group.

According to a large number of previous clinical research results, it has also been shown that compared with conventional neoadjuvant chemoradiotherapy, neoadjuvant chemoimmunotherapy for esophageal squamous cell carcinoma has a relatively lower pathological complete response (pCR) rate, but a relatively longer long-term survival time. Therefore, future studies on chemoimmunotherapy can focus on how to improve the pCR rate of the treatment while ensuring the long-term survival time of chemoimmunotherapy. Meanwhile, subsequent studies can also continue to explore the efficacy of different immunotherapeutic agents in neoadjuvant therapy, while identifying treatment-related biomarkers to better achieve individualized treatment.

In this Research Topic, the article by Zhang et al. showed that patients who achieved pathological complete response (pCR) after neoadjuvant therapy had a higher 3-year recurrence-free survival rate. This also suggests that it is crucial to explore ways to improve the pCR rate of chemoimmunotherapy. Meanwhile, Li et al. explored the impact of neoadjuvant immunotherapy combined with chemotherapy or chemoradiotherapy on postoperative safety in locally advanced esophageal squamous cell carcinoma. Chen et al. explored biomarkers for tumor regression grade in esophageal squamous cell carcinoma patients after neoadjuvant chemoradiotherapy. This also provides a basis for selecting appropriate patients and implementing individualized treatment in the later stage.

In general, patients with locally advanced esophageal squamous cell carcinoma can significantly benefit from neoadjuvant therapy. However, how to select treatment regimens, the advantages and disadvantages of different regimens, and the choice of individualized treatment for patients remain the goals to be studied in the future.
